# Assessment of longitudinal changes in strain using DENSE in patients with myocardial infarction

**DOI:** 10.1186/1532-429X-16-S1-P187

**Published:** 2014-01-16

**Authors:** Christie McComb, David Carrick, Rosemary Woodward, John D McClure, Aleksandra Radjenovic, John Foster, Colin Berry

**Affiliations:** 1 BHF Glasgow Cardiovascular Research Centre, Glasgow, UK; 2 Clinical Physics, NHS Greater Glasgow and Clyde, Glasgow, UK; 3 Cardiology, Golden Jubilee National Hospital, Glasgow, UK; 4 MRI, Golden Jubilee National Hospital, Glasgow, UK

## Background

Myocardial infarction (MI) causes contractile dysfunction in the affected tissue, which can be assessed by using DENSE (Displacement ENcoding with Stimulated Echoes) to quantify myocardial strain[1,2]. The aim of this study was to investigate changes in strain revealed by DENSE between the occurrence of MI and a 6 month follow-up, and the relationships with other clinical measures.

## Methods

50 male patients (age 56 ± 10 years) underwent CMR on a 1.5T Siemens Avanto within 7 days of MI, and 47 returned for a follow-up scan after 6 months. The protocol included cine, DENSE (2D) and late gadolinium enhancement (LGE) imaging. Cine images were used to assess cardiac function by calculating LV ejection fraction (LVEF) and end-systolic volume (LVESV). DENSE and LGE were compared using a single mid-ventricular short-axis slice, which was analysed both as a whole slice and after division into 6 AHA segments. The percentage of each segment which contained LGE was calculated using a threshold of mean+5SD of remote myocardium intensity. DENSE images were analysed to obtain a value for peak circumferential strain (Ecc). Segments in the baseline scans were grouped according to the extent of LGE (non-infarcted, <50% infarcted, >50% infarcted), and the change in peak Ecc between baseline and follow-up was evaluated using a one-way ANOVA with Tukey's post-hoc test. Individual patients were compared directly, and the correlations between change in strain and (i) change in LGE (segments) and (ii) change in cardiac function (slices) were assessed.

## Results

**Figure 1:**
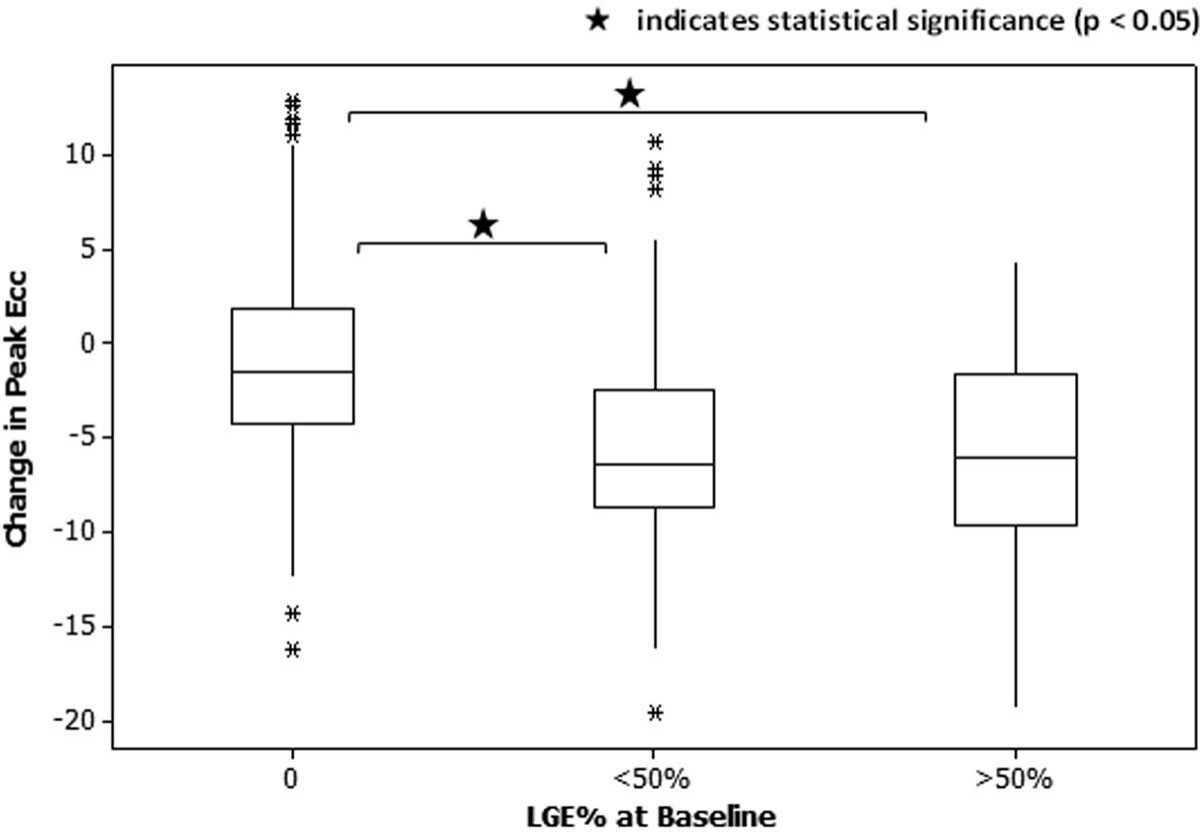


**Figure 2:**
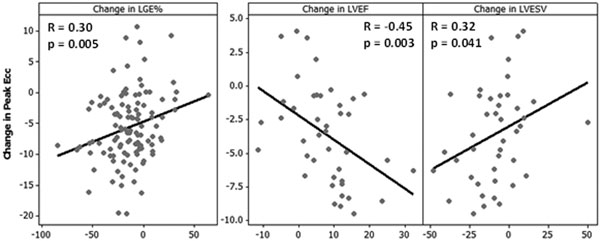


Diagnostic images were obtained for 50 patients at baseline, and for 43 patients at follow-up. The results of the group comparisons and the individual patient comparisons are illustrated in Figure 1 and Figure 2 respectively.

## Conclusions

Strain recovery was disclosed by DENSE in infarcted tissue at 6 months post-MI. An increase in peak Ecc at follow-up is associated with a reduction in LGE, and improvement in LVEF and LVESV. Further work is required to take infarction in adjacent segments and slices into account, as this may provide a more precise insight into the relationship between myocardial strain, LGE and cardiac function.

## Funding

N/A.
